# Detect AD Patients by Using EEG Coherence Analysis

**DOI:** 10.1155/2014/236734

**Published:** 2014-02-10

**Authors:** Ming-Chung Ho, Tsung-Ching Chen, Chin-Fei Huang, Cheng-Hsieh Yu, Jhih-Ming Chen, Ray-Ying Huang, Hsing-Chung Ho, Chia-Ju Liu

**Affiliations:** ^1^Department of Physics, National Kaohsiung Normal University, Kaohsiung 824, Taiwan; ^2^Graduate Institute of Science Education, National Kaohsiung Normal University, Kaohsiung 824, Taiwan

## Abstract

The purpose of this study is to discriminate mild Alzheimer's disease (AD) patients from the normal aging. The EEG coherence was applied to analyze the data from auditory oddball paradigm to discriminate the differences of corticocortical connections between mild AD patients and healthy subjects. The results showed that the lower values of coherence were performed in mild AD patients than in the normal aging subjects, especially in theta band. The implications and suggestions are shown in this study.

## 1. Introduction

Event-related brain activity remains incompletely understood in terms of the nature of the networks of corticocortical connections during normal aging [1]. Recently, the graph analysis was based upon matrices of synchronization, which analyzed the brain networks of aging applied in electroencephalography (EEG) studies [2].

Dementia which is an age-related disease affects 17–25 million people worldwide [3]. With the rapidly increased population of old people, the dementia patients also increased rapidly. Alzheimer's disease (AD) is the leading cause of dementia. And the early stage of AD is mild Alzheimer's disease (AD). Hence, to diagnose mild AD earlier is very important to help patients to change to AD patients.

Event-related potentials (ERPs) have the benefit of being a noninvasive technique with good time resolution and it is a biological marker for early detection of AD used to discriminate between normal aging and AD [4]. However, while ERPs components were often able to discriminate between patients and control groups, they have not proved sufficiently sensitive for the diagnosis of individual patient [5]. Now, during cognitive task, EEG coherence herein provides additional sources of information about the corticocortical potential interactions [6].

Previous researches suggested that patterns of high coherence between EEG signals recorded on different scalp locations have functional significance and are correlated with different kinds of cognitive information processing [7]. In clinical studies, the measure of coherence has been used to investigate the connectivity between the various cortical areas of Alzheimer patients. Studies comparing normal older adults to patients with AD have reported further reductions in interhemispheric alpha band (8–12 Hz) coherence between occipital sites and in temporo-parietooccipital areas [8]. Besides, the functional connectivity of age-related changes is still poorly understood [9] in auditory ERP studies. Therefore, more information about age-related changes in ERP oscillations is needed, especially in the time-frequency domain [10]. Therefore, this study analyzed the differences of the coherence between the frontal and tempoparietal regions about mild AD and healthy controls upon the application of auditory oddball paradigm. Furthermore, this study also investigates the components of ERPs in brain networks by ERPCOH analysis.

## 2. Methods

This study recruited 15 healthy university students (mean = 21, S.D. = 1.13), 15 normal aging (mean = 72, S.D. = 10.47), and 16 AD patients (mean = 80, S.D. = 9.61). None of the participants reported hearing loss or neurological or psychological problems, and all were naive to electrophysiological studies. Participants gave informed consent. At the time of investigation no patients were taking any prophylactic medication or receiving nonpharmacological treatments.

Standard (2000 Hz) and target (1000 Hz) auditory stimuli were presented binaurally over headphones to each participant with a duration of 1000 msec. The target tone occurred regularly with a 0.20 probability. The rise and fall time of each tone was 5 msec. During the recording session, participants sat in a chair, in a brightly illuminated room, in a relaxed position. They were instructed to sit with open eyes, to follow the stimuli carefully, and then try to detect target tone of 1000 Hz. All participants achieved minimum 95% accuracy.

EEG was recorded with the SynAmps/SCAN 4.4 hardware and software (NeuroScan, Inc., Herndon, VA) from 32 tin electrodes mounted in a commercial electro-cap (ElectroCap International, Eaton, OH), and the electrode impedance was always kept below 5 kΩ. The common reference electrodes for EEG measurements were placed on mastoids behind the ears. Stimulus presentation was generated by Neuroscan Stim 3.3 Software. For monitoring eye movements an electrode placed at the external canthi of the left eye was used. EEG channels were continuously digitized at a rate of 10000 Hz by a SynAmp amplifier. The signal was analog-filtered (0.1–200 Hz), A/D converted with a sampling rate of 10000 Hz and 14 bit precision, and digitally filtered in the range 0.1–50 Hz.

The EEG data were recoded from 30 electrodes positioned according to the International 10–20 system (i.e., FP1, FP2, F7, F3, Fz, F4, F8, FT7, FC3, FCz, FC4, FT8, T3, C3, Cz, C4, T4, TP7, CP3, CPz, CP4, TP8, T5, P3, Pz, P4, T6, O1, Oz, and O2), with analog-to-digital (A/D) conversion with a sampling rate of 1,000 Hz. Data from single-trial epochs exhibiting excessive movement artifacts (>90 *μ*V) were rejected. After removal of artifacts, between 28 and 30 target events remained for each participant, from which a mean was calculated. The EEG data was recorded in a sound-attenuated room. During auditory oddball conditions, subjects kept their eyes closed.

EEG coherence represents the covariance of the EEG spectral activity at two electrode locations and the temporal synchronization or functional coupling of the two cortical populations generating the scalp EEG data collected by the paired electrodes [11]. It can be a measure of temporal synchronization of the EEG signals recorded on pairs of electrodes. In reality, the EEG coherence analysis just captures the linear component of the functional coupling of the paired EEG oscillations. And the analysis of EEG coherence is the most common approach for the study of functional coupling of EEG oscillations in aging [8]. Now, coherence was calculated by the following equation: Coh_*xy*_(*λ*) = |*R*_*xy*_(*λ*)|^2^ = |*f*_*xy*_(*λ*)|^2^/*f*_*xx*_(*λ*)*f*_*yy*_(*λ*), where *f* denotes the spectral estimate of two EEG signals *x* and *y* for a given frequency bin(*λ*). The numerator contains the cross-spectrum for *x* and *y*  (*f*_*xy*_), while the denominator contains the respective autospectra for *x*  (*f*_*xx*_) and *y*  (*f*_*yy*_). This equation returns a real number between 0 (no coherence) and 1 (max coherence). In this study, the EEG coherence was computed on CP3 and F4 pairs of electrodes interest to obtain the two electrodes. Coherence values were computed about the younger, normal aging, and Mild AD.

Time-frequency representations of the ERPCOH data were used in the theta (4–7 Hz), alpha-1 (7–10 Hz), alpha-2 (10–13 Hz), beta-1 (13–20 Hz), beta-2 (20–30 Hz), and gamma (30–50 Hz) bands. Early and late components of ERPs were N1 (80–140 ms) and P3 (280–450 ms) for the auditory stimuli.

In this study, the time-frequency transformation provides an estimation of the average time-varying ERPCOH of the signal in each frequency band. If *x* and *y* denote two signals from two electrode sites, then the ERPCOH can be defined as

(1) $$\begin{array}{c} \text{ERPCO}{\text{H}}^{x,y}\left(f,t\right)=\frac{1}{n}\sum_{i=1}^{n}\frac{F_{i}^{x}\left(f,t\right)F_{i}^{y}{\left(f,t\right)}^{*}}{\left|F_{i}^{x}\left(f,t\right)F_{i}^{y}\left(f,t\right)\right|}, \end{array}$$

where *n* denotes the number of trials, *F*_*i*_^*x*^(*f*, *t*) the spectral estimate of trial *i* at frequency *f* and time *t* on the signal *x*, and *F*_*i*_^*y*^(*f*,*t*)^*^ the complex conjugate of *F*_*i*_^*y*^(*f*, *t*). The magnitude of ERPCOH varied between 0 (absence of synchronization) and 1 (perfect synchronization), which is normalized for comparative purposes. Conditions of high ERPCOH correspond to stronger connectivity, which relates to neural synchronization. A sliding temporal Hanning window of 256 points was used to obtain 1 Hz frequency resolution and 4 ms time resolution. To avoid possible edge effects and artifactual contamination (eye movement or muscle activity) for the ERPCOH measurement, the frequency band used in the time-frequency domain was 3.9–50 Hz. The ERPCOH analysis was conducted using EEGLAB v9.0 [10] under Matlab 7.

The ERPCOH values were used to construct a 30 × 30 matrix of all possible pair-wise combinations. The matrix was transformed into a binary (unweighted) graph using a threshold value, which was chosen for each graph using a fixed mean degree. The efficiency of brain networks was then calculated.

The two main efficiency parameters, which were introduced by Nunez [11], were global efficiency, *E*_glob_, and local efficiency, *E*_loc⁡_. Let *N* be the set of all nodes in the network, *n* the number of nodes, and (*i*, *j*) a link between nodes *i* and *j*, (*i*, *j* ∈ *N*). The global efficiency *E*_glob_ of the network can be defined as

(2) $$\begin{array}{c} E_{\text{glob}}=\frac{1}{n}\sum_{i\in N}\frac{1}{n-1}\sum_{j\in N,\;j\neq i}\frac{1}{d_{ij}}, \end{array}$$

where *d*_*ij*_ denotes the shortest path length (distance) between nodes *i* and *j*. The local efficiency *E*_loc⁡_ of the network can be defined as

(3) $$\begin{array}{c} E_{\mathrm{loc}}=\frac{1}{n}\sum_{i\in N}\frac{1}{k_{i}\left(k_{i}-1\right)}\sum_{j,\;h\in N,\;j\neq i}a_{ij}a_{ih}\frac{1}{d_{jh}\left(N_{i}\right)}, \end{array}$$

where *k*_*i*_ ( = ∑_*j*∈*N*_ *a*_*ij*_) denotes the degree of a node *i* and *a*_*ij*_ is the connection status between *i* and *j*: *a*_*ij*_ = 1 when link (*i*, *j*) exists (when *i* and *j* are neighbors); *a*_*ij*_ = 0 otherwise (*a*_*ii*_ = 0  for  all  *i*), and *d*_*jh*_(*N*_*i*_) denotes the length of the shortest path between *j* and *h* that contains only the neighbors of *i*. Global efficiency and local efficiency are involved in characterizing functional integration and segregation among cortical areas, respectively [12]. Global efficiency assesses the ability for information transfer between any two nodes via multiple parallel paths, while local efficiency assesses the ability of information transfer through the entire subgraph of a node's connection [13]. Since the structure of the graph can be biased by the number of edges between nodes, a statistical measure, equal degree *K*, was calculated for the graph. Therefore, the threshold value was chosen such that each analyzed graph had a fixed mean degree (*K* = 8), suggesting that each subject also has the same cost value, which is 0.277 in this study. We then computed *E*_glob_ and *E*_loc⁡_. The *E*_glob_ and *E*_loc⁡_ values were compared with the same nodes and degrees of 50 random graphs (*E*_glob-rand_ and *E*_loc-rand_) using the ratios *ε*_glob_ = *E*_glob_/*E*_glob-rand_ and *ε*_loc⁡_ = *E*_loc⁡_/*E*_loc-rand_. Additionally, the global efficiency of SWN is less than the corresponding efficiency of random networks, leading to normalized global efficiency *ε*_glob_ < 1. Moreover, an SWN in local efficiency is higher than that of a random network, as described by normalized local efficiency *ε*_loc⁡_ ≫ 1 [14]. Statistical results were obtained with analysis of variance (ANOVA) using SPSS 12.0.

## 3. Results and Discussion

**Figure 1:**
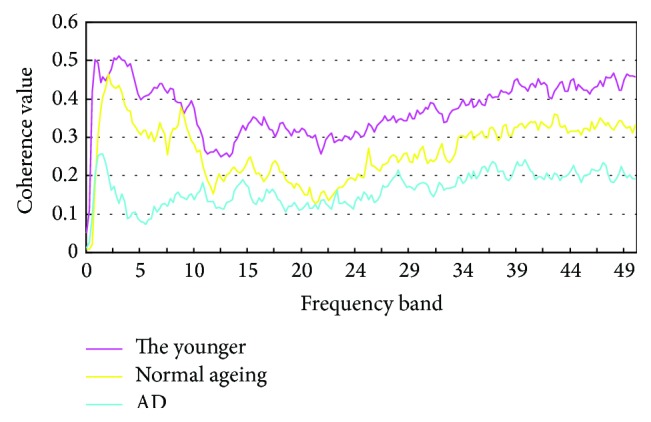
Difference of coherence in frequency between groups.

The result in Figure 1 shows that the mean values of the younger and normal aging are almost the same, but the mean value of mild AD is significantly different. The value of AD is lower than the normal aging and the younger, especially, between frequency 4–7 Hz. It reveals that the coherence value of the AD is lower than the normal aging and the younger from 0.1 to 50 Hz frequency. The trend reveals AD patients have decreased EEG coherence and it is extremely significant difference.

**Figure 2:**
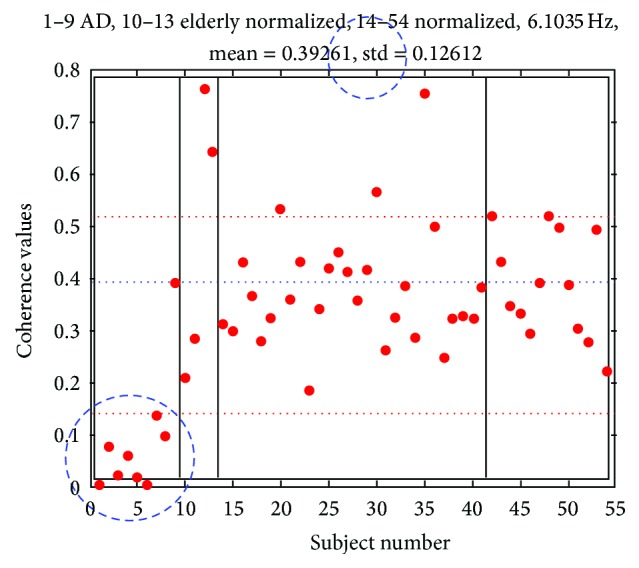
The effect of individual discrimination and the AD patients all have lower coherence value between 0 to 0.1.

Then, this study is compared with coherence value of three groups from different frequency bands in Figure 2. The results show that the effect of individual discrimination is the most significant in theta band (frequency 4–7 Hz).

**Figure 3:**
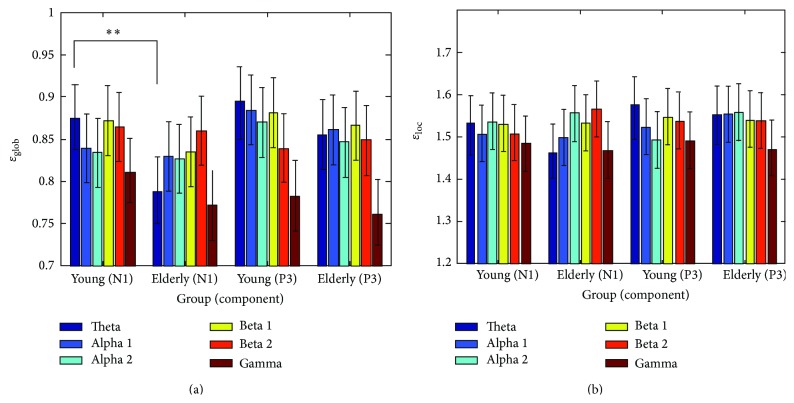
(a) Mean *ε*_glob_ and (b) mean *ε*_loc⁡_ values from ERPCOH were obtained for the young and elderly in various frequency bands when responding to the target stimuli. The vertical bars indicate 95% confidence intervals. Statistically significant differences are marked with symbols representing significance levels (^**^ *P* < .05).

In Figure 2, the effect of individual AD patients could be discriminated, but the differences between younger and elders could not be discriminated. Hence, this study used ERPCOH analysis to demonstrate the differences between younger and elders. In Figure 3, the (normalized) global efficiency was lower in the elderly (*F*[1,28] = 11.340, *P* = .001) than in the young, whereas the values of early theta band for the two groups were significantly different for the target stimuli (*P* < .001). The (normalized) local efficiency was higher in the elderly (*F*[1,28] = 4.929, *P* = .027, see Figure 3(b)) than in the young, whereas no differences were found in any of the frequency bands for the two groups.

To sum up, EEG coherence from the waveform of all subjects revealed that the connection between temporoparietal and right frontal lobe is important, especially in theta band. Decreased coherence of AD patients was found for alpha frequency bands and in some cases also for the beta band [8]. Besides, this study indicates that the global efficiency of brain networks was decreased in the elderly when responding to the target stimulus, especially in the early theta band, providing evidence that processing efficiency declines with aging. Additionally, these decreases in global efficiency were correlated with reduced connections, which is likely to reflect a prolonged mean reaction time in auditory information processing for the elderly. The pattern of changes in the elderly probably reflects a tendency of an aging-related shift toward regular networks [15].

## 4. Conclusion

This study analyzes the EEG coherence by using the response from auditory oddball paradigm to know well the differences of corticocortical connections between AD patients and healthy controls. EEG coherence is better indicator to discriminate AD and the normal, especially in the theta band. Further, a major finding from ERPCOH is also shown that the theta band is the primary characteristic of normal aging.

The result may suggest that AD patients have problem in connection between frontal and temporoparietal lobe while performing attention and memory task. Finally, the individual discrimination in Alzheimer's disease is efficient by the method of EEG coherence.
